# Circ_0003945: an emerging biomarker and therapeutic target for human diseases

**DOI:** 10.3389/fonc.2024.1275009

**Published:** 2024-04-22

**Authors:** Xiaofei Zhang, Li Ma, Li Wan, Haoran Wang, Zhaoxia Wang

**Affiliations:** ^1^ Cancer Medical Center, The Second Affiliated Hospital of Nanjing Medical University, Nanjing, China; ^2^ Department of Oncology, The Affiliated Huai’an No.1 People’s Hospital of Nanjing Medical University, Huai’an, China; ^3^ Division of Spine Surgery, Department of Orthopedics, Tongji Hospital, Tongji University School of Medicine, Shanghai, China

**Keywords:** cancer, circ_0003945, ceRNA, biomarker, therapeutic target

## Abstract

Due to the rapid development of RNA sequencing techniques, a circular non-coding RNA (ncRNA) known as circular RNAs (circRNAs) has gradually come into focus. As a distinguished member of the circRNA family, circ_0003945 has garnered attention for its aberrant expression and biochemical functions in human diseases. Subsequent studies have revealed that circ_0003945 could regulate tumor cells proliferation, migration, invasion, apoptosis, autophagy, angiogenesis, drug resistance, and radio resistance through the molecular mechanism of competitive endogenous RNA (ceRNA) during tumorigenesis. The expression of circ_0003945 is frequently associated with some clinical parameters and implies a poorer prognosis in the majority of cancers. In non-malignant conditions, circ_0003945 also holds considerable importance in diseases pathogenesis. This review aims to recapitulate molecular mechanism of circ_0003945 and elucidates its potential as a diagnostic and therapeutic target in neoplasms and other diseases.

## 1 Introduction

Cancer represents a serious public health issue that affects people all around the world (1). Various etiological factors and socioeconomic elements including population aging exacerbate the cancer burden and contributing to an increase in cancer-related fatalities (2). Despite the availability of cutting-edge medical diagnostics and therapeutics, recurrence and metastasis still pose dramatic barriers to achieving long-term survival in patients (3–5).

**Figure 1:**
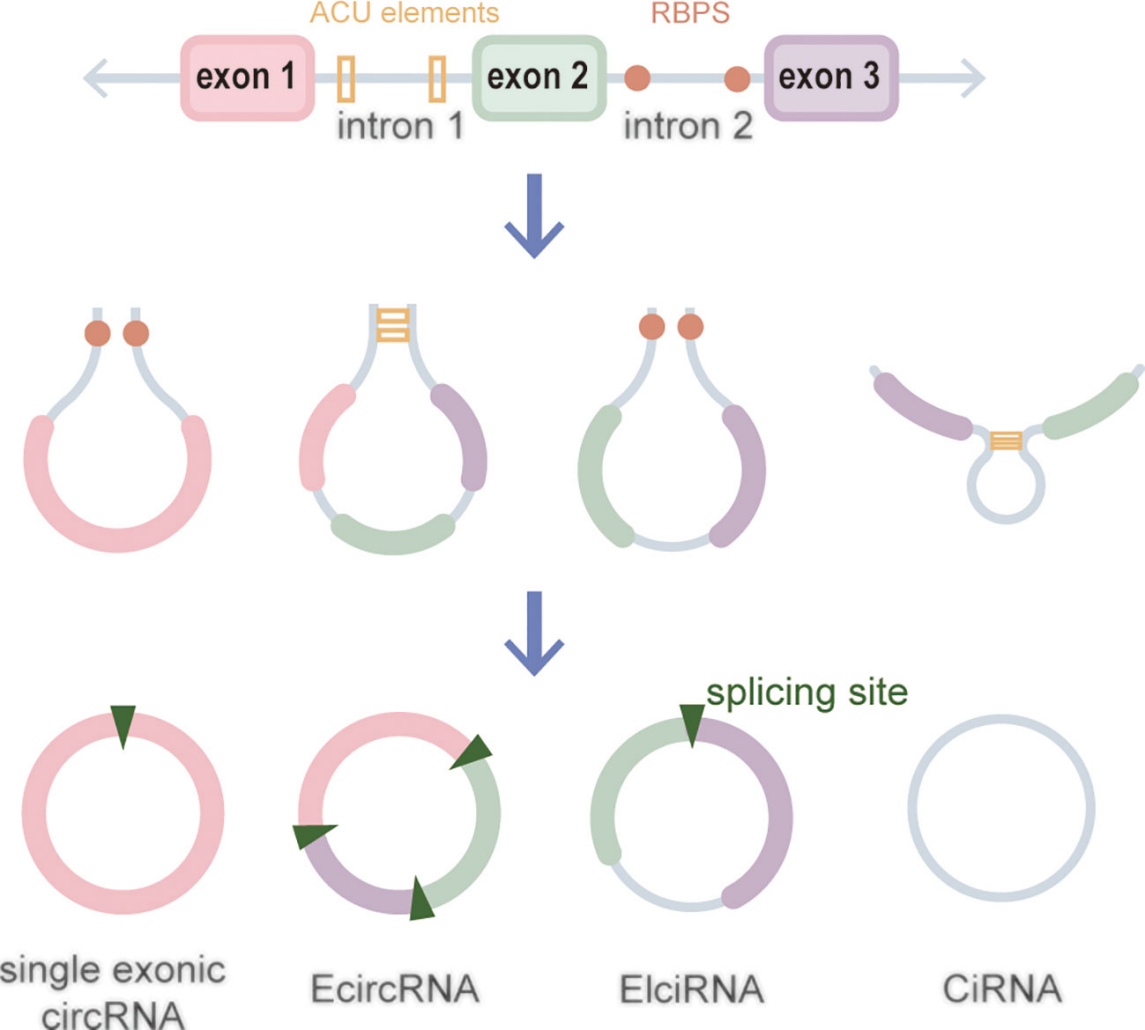
Biogenesis of circRNAs.

**Figure 2:**
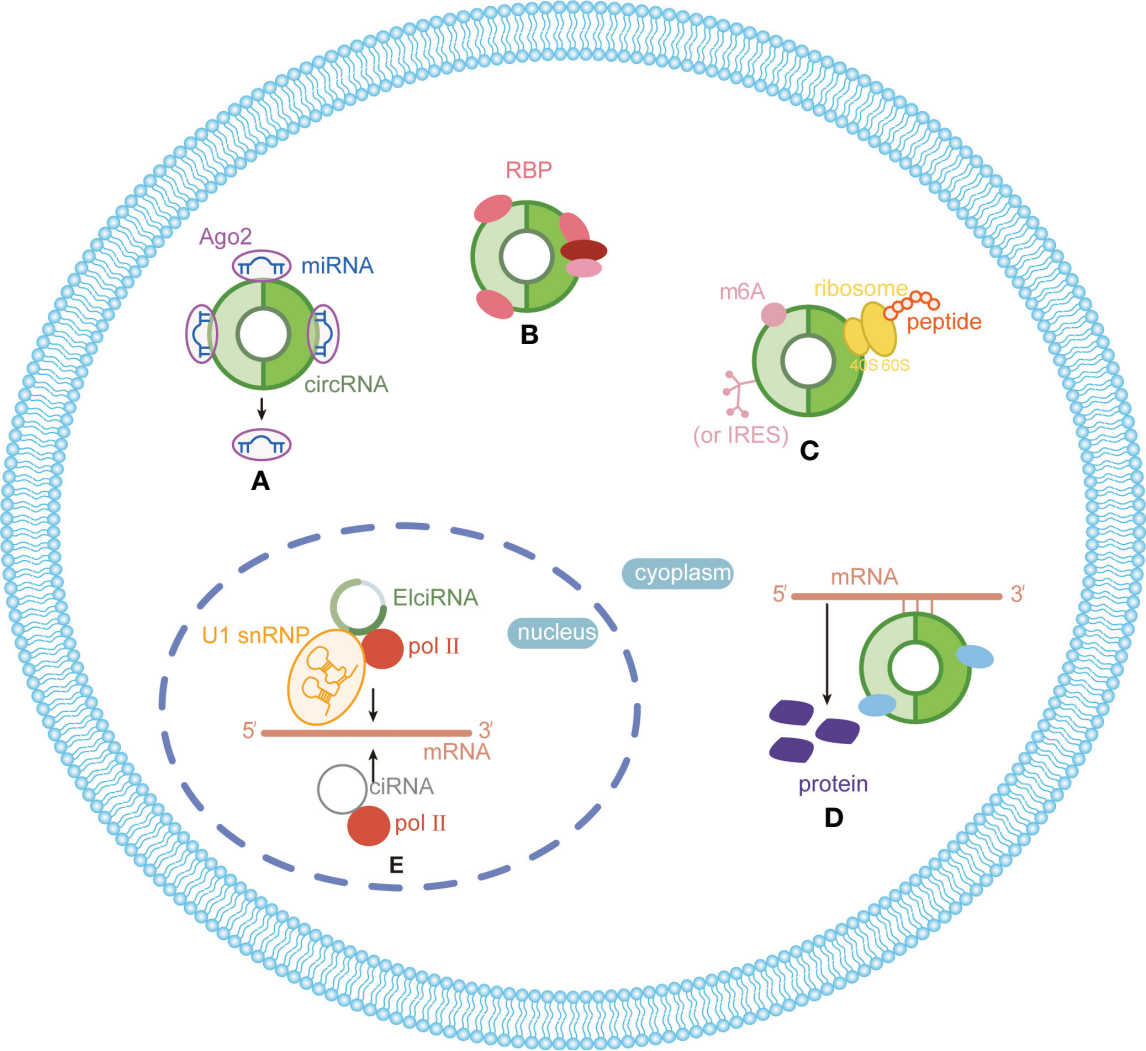
Functions and mechanisms of circRNAs in eukaryotes. **(A)** Acting as miRNA sponge; **(B)** acting as protein sponge and scaffold; **(C)** acting as templates for translation; **(D)** maintaining mRNA stability and regulate translation; **(E)** regulating transcription.

CircRNAs, a new class of non-coding RNA (ncRNA) distinct from traditional linear RNAs, are characterized by their covalently closed, continuous loop structures (6). First identified in plants viroids in 1976 (7), circRNAs were subsequently detected in eukaryotic cells in 1979 (8). The discovery that the hepatitis delta virus (HDV) has a single-stranded circRNA molecule marked it as the first known animal virus with a circRNA genome in 1986 (9). Specifically, circRNA is generated by the RNA polymerase II (pol II)–mediated back-splicing of pro-mRNA (10). Back-splicing forms a stable closed-loop structure devoid of 3’ or 5’ end. It restrains the exonuclease-mediated degradation due to its covalent bonding of downstream and upstream splice-donor sites (11). The nucleotide (nt) length of circRNAs typically ranges from several hundred to a few thousand, and it generally composed of one to five exons (12). Depending on their splicing sources, circRNAs can be categorized into single-exonic circRNAs, exonic circRNAs (EcircRNAs), intronic circRNAs (CiRNAs), exon–intron circRNAs (EIciRNAs), even tRNA intron cirRNAs (tricRNAs) according to their splicing sources (**Figure 1**) (13–15). CircRNAs are widely expressed in eukaryotes, where EcircRNAs are predominantly localized in the cytoplasm, while EcircRNAs and EIciRNAs are primarily nucleus (16, 17). In summary, circRNAs demonstrates tissue-specific localization and evolutionary conservation, stably persisting in intricate intracellular and extracellular environments, holding promise as ideal tumor biomarkers (18, 19). CircRNAs perform various biological functions (**Figure 2**): (1) as endogenous RNAs (ceRNA), circRNAs act as molecular sponges, impeding the microRNA (miRNA)–mediated repression of target genes (20, 21). (2) CircRNAs serve as molecular sponges for proteins, particularly RNA-binding proteins, regulating the transcription or translation of downstream target genes (22, 23). (3) CircRNAs function as protein scaffolds facilitating interactions between specific proteins (16, 24). (4) CircRNAs possess translational capacity, producing peptides that exert biological functions (25–27). (5) CircRNAs maintain the stability of mRNA and regulate its translation, facilitating or inhibiting the respective translation processes (22, 28). (6) EIciRNAs and CiRNAs have been identified to be transcription regulators. EIciRNA locating in the nuclear can interact with the U1 small nuclear ribonucleoprotein (snRNP) and bind to RNA pol II, enhancing the transcription of their host genes. CiRNAs also modulate RNA pol II–mediated transcription, exerting a cis-regulatory effect on upstream genes (29). CircRNAs can directly bind with nuclear transcription factors (TFs) to regulate their activity and function (30). Additionally, the transcription of some circRNAs might occur independently of host genes, regulated by TFs, as opposed to the general assumption of deriving from host transcript splicing (31). For instance, a set of circRNAs with transcription activation levels higher than those of host genes, identified as transcriptionally activated to a higher level than the host genes (TAH)-circRNAs, exhibited more TFs occupancy in their regulatory regions. Certain TFs, especially the validated super enhancer (SE) FOXA1 validated already, directly regulated the transcription of TAH-circRNAs (32, 33). A novel genetic unit, the intragenic regulon (iRegulon), also differently regulates the expression of linear and circular RNA products, thereby manifesting distinct biological functions (30). To date, circRNAs have been established as participants in variety of physiopathological processes (14).

**Figure 3:**
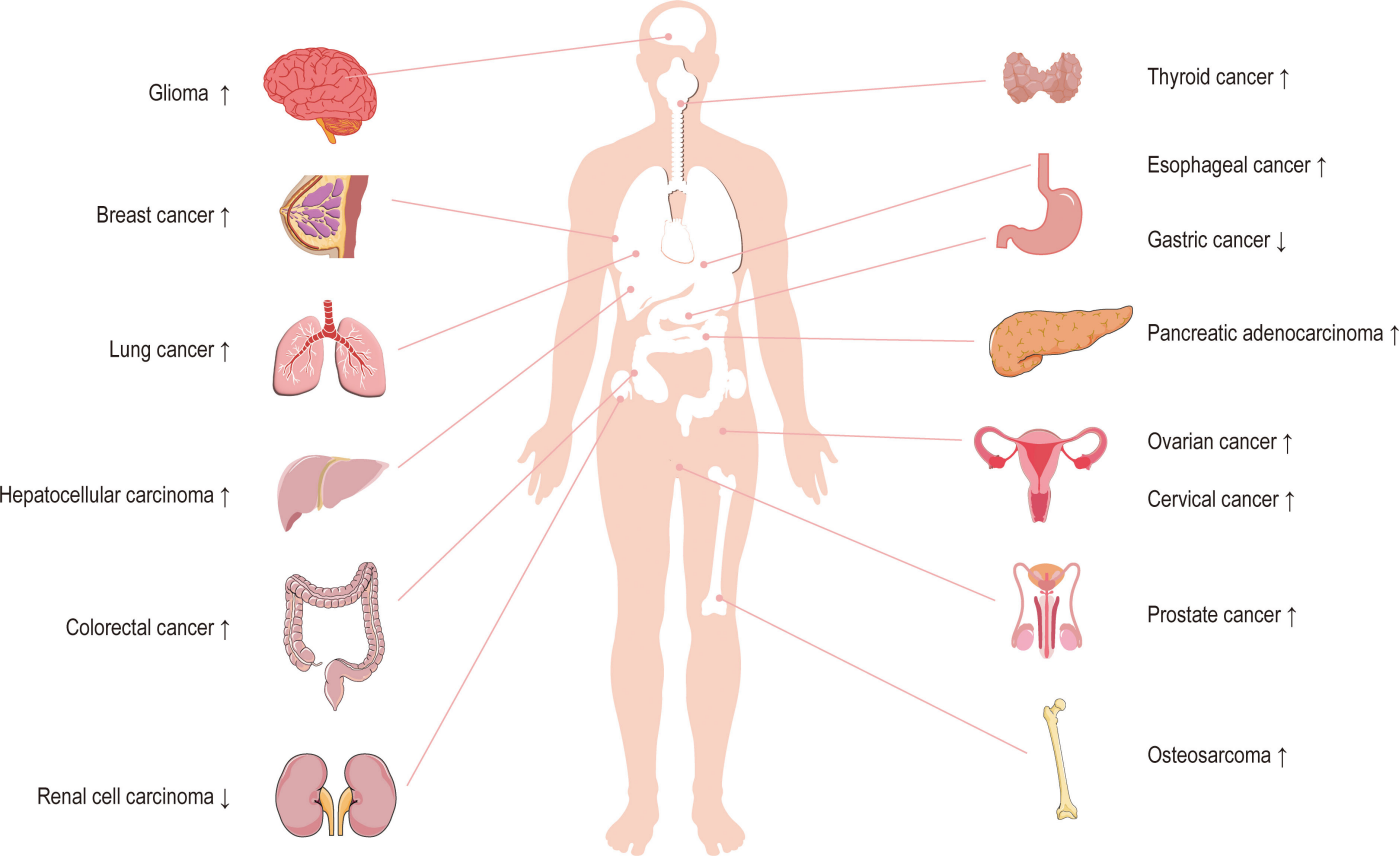
The expression of circ_0003945 in human tumors.

Circ_0003945 (circBase ID: hsa_circ_0003945) was located on chr9: 33953282–33956144, with a total length of 258 nt. It was formed by back-spliced of the 11 and 12 exons of the ubiquitin-associated protein 2 (UBAP2) and aliased hsa_circ_0001846 (34), hsa_circ_0001850 (35), hsa_circ_0003141 (36), hsa_circ_0003496 (37), hsa_circ_0007367 (38), hsa_circ_0008344 (39), and hsa_circ_0086735 (40) (**Table 1**). For the sake of narrative clarity in this text, it will be uniformly referred to as circ_0003945. Studies have confirmed that circ_0003945 showed resistance to digestion by ribonuclease R (RNase R), whereas the corresponding linear transcript was considerable diminished after RNase R treatment, underscoring the stability of its covalently closed-loop structures (42, 43). Circ_0003945 have been associated with a variety of diseases, including various neoplasms and non-malignant conditions such as microcirculatory perfusion (44, 45), diabetic retinopathy (DR) (35), osteoarthritis (OA) (46), preeclampsia (PE) (47), and milk fat metabolism (48). Noteworthy, circ_0003945 was highly overexpressed and linked to poor prognosis, including in glioma (49), thyroid cancer (TC) (36), esophageal cancer (EC) (50), non-small cell lung cancer (NSCLC) (51), breast cancer (BC) (34), hepatocellular carcinoma (HCC) (52), pancreatic cancer (53), colorectal cancer (CRC) (54), ovarian cancer (OC) (55), cervical cancer (CC) (56), prostate cancer (57), and osteosarcoma (58) (**Figure 3**). However, it is noteworthy that the expression of circ_0003945 in gastric cancer (GC) (59) and renal cell carcinoma (RCC) (60) were lower compared to normal tissue, which also suggested a more favorable prognosis. Additionally, circ_0003945 was implicated in the regulation of tumor cell proliferation, migration, invasion, apoptosis, drug resistance, and radio resistance (**Table 2**) and correlated with the clinicopathological characteristics of tumor patients (**Table 3**). This review will synthesize the potential molecular mechanisms by which circ_0003945 driven tumorigenesis and its clinical significance in human diseases.

**Table 1:** Alias of circ_0003945 and different splicing methods from UBAP2 gene in diseases.

CircRNA ID	Position	Genomic length (nt)	Spliced sequence length (nt)	Involving disease	Ref.
hsa_circ_0001846 (alias hsa_circ_1335)	chr9:33944362-33956144	11782	747	BC, prostate cancer, OA	(34)
hsa_circ_0001850 (alias hsa_circ_1782)	chr9:33960823-33973235	12412	278	DR	(35)
hsa_circ_0003141	chr9:33953282-33973235	19953	536	TC	(36)
hsa_circ_0003496	chr9:33948371-33953472	5101	404	Osteosarcoma, PE	(37)
hsa_circ_0003945	chr9:33953282-33956144	2862	258	HCC	(41)
hsa_circ_0007367	chr9:33948371-33956144	7773	472	Pancreatic cancer, cardiogenic shock	(38)
hsa_circ_0008344	chr9:33935836-33941860	6024	254	Glioma	(39)
hsa_circ_00086735	chr9:33986757-34017187	30430	561	CRC	(40)

**Table 2:** Functional characterization of circ_0003945 in tumors and non-tumor conditions.

Tumor types	Expression	Role	Assessed tumor cell lines	Function roles	Animal studies	Related genes	Ref.
Glioblastoma	Upregulate	Oncogene	U87, U251	Proliferation Migration Invasion Apoptosis	–	–	(49)
Glioma	Upregulate	Oncogene	U251, LN229	Proliferation Apoptosis Radio resistance	Male BALB/c nude mice: tumor volume, weight	miR-433-3p, RNF2	(61)
Glioma	Upregulate	Oncogene	U251	Proliferation Migration Invasion Apoptosis	Female BALB/c mice: tumor volume, weight	miR-1205, miR-382, GPRC5Ak	(62)
Glioma	Upregulate	Oncogene	U251, A172	Proliferation Migration Invasion Angiogenesis	Male BALB/c nude mice: tumor volume, weight	miR-638, SZRD1	(39)
Papillary thyroid cancer	Upregulated	Oncogene	TPC-1, IHH-4	Proliferation Invasion Apoptosis	–	miR-370-3p, PI3K/Akt and Wnt pathways	(63)
Esophageal squamous cell carcinoma	Upregulated	Oncogene	TE-9	Proliferation Migration Invasion	BALB/C nude mice: tumor volume, weight	miR-422a, Rab10	(50)
Lung adenocarcinoma	Upregulated	Oncogene	A549	Proliferation Invasion Metastasis Apoptosis	–	miR-339-5, miR-96-3p, miR-135b-3p, JNK-MAPK pathway, Rac1-FAK pathway	(51)
Non-small cell lung cancer	Upregulated	Oncogene	NCI -H1299, A549	Proliferation Migration Invasion Chemo resistance	Female BALB/c nude mice: tumor volume, weight	miR-3182, KLF4	(43)
Triple-negative breast cancer	Upregulated	Oncogene	BT-20, MDA-MB-231	Proliferation Migration Metastasis Apoptosis	Female BALB/c nude mice: tumor volume, weight, lung metastasis	miR-661, MTA1	(34)
Triple-negative breast cancer	Upregulated	Oncogene	BT-549/DDP, MDA-MB-436/DDP	Proliferation Migration Invasion Apoptosis Cisplatin resistance	–	miR-300, ASF1B, PI3K/AKT/mTOR pathway	(64)
Luminal breast cancer	Upregulate	Oncogene	MCF7, ZR-75-1	Proliferation Apoptosis Tamoxifen resistance	–	miR-1296-5p, STAT1,	(65)
Hepatocellular carcinoma	Upregulate	Oncogene	Huh-7, Hep3B2.1-7	Proliferation Invasion Apoptosis EMT	BALB/c nude mice: tumor volumes, weights	miR-1827, UBAP2	(52)
Hepatocellular carcinoma	Upregulated	Oncogene	HA22T, Huh7	Proliferation Migration Invasion Metastasis	male nude mice: tumor volume, weight, lung metastasis	miR-194-3p	(42)
Hepatocellular carcinoma	Upregulate	Oncogene	MHCC97H, HCCLM3, Li-7	Proliferation Migration	Male BALB/c nude mice: tumor volume, weight	miR-34c-5p, LGR4, wnt/β-catenin pathway	(41)
Hepatocellular carcinoma	Upregulated	Oncogene	MHCC97H, HCCLM3	Proliferation Migration Invasion EMT Apoptosis	NOG mice: tumor volume, weight	miR-1294, c-Myc	(66)
Hepatocellular carcinoma	Upregulated	Oncogene	MHCC-97H, Huh-7	Migration Invasion	BALB/c nude male mice: tumor volume, weight, lung metastasis	CXCL11, miR-4756, IFIT1/3	(67)
Pancreatic adenocarcinoma	Upregulated	Oncogene	–	Immune infiltration	–	miR-494, CXCR4, ZEB1, SDC1, HIF1A	(53)
Pancreatic ductal adenocarcinoma	Upregulated	Oncogene	AsPC-1, PANC-1	Proliferation Migration Invasion	BALB/c nude mice: tumor volume, weights	Mir-6820-3p, YAP1	(38)
Colorectal cancer	Upregulated	Oncogene	SW620, HCT116	Proliferation Invasion Migration	–	miR-199a, VEGFA	(54)
Colorectal cancer	Upregulated	Oncogene	HCT116, SW480	Proliferation Invasion Migration Metastasis	Female BALB/c nude mice: tumor size, weight, lung metastasis	miR-582-5p, FOXO1	(40)
Ovarian cancer	Upregulated	Oncogene	OVCAR3, HO8910	Proliferation Migration	–	miR-144, CHD2	(55)
Ovarian cancer	Upregulated	Oncogene	OVCAR-3, ES-2	Proliferation Apoptosis	–	miR-382-5p, PRPF8	(68)
Cervical cancer	Upregulated	Oncogene	HeLa, SiHa	Proliferation Migration Invasion Metastasis EMT Apoptosis	BALB/c nude mice: tumor volumes, weight, lung metastasis	miR-361-3p, SOX4	(56)
Prostate cancer	Upregulated	Oncogene	LNCaP, V16A, DU145, PC-3	Proliferation	–	miR-1244, MAP3K2, MAPK pathway	(57)
Osteosarcoma	Upregulate	Oncogene	MG63, U2OS	Proliferation Apoptosis	BALB/c athymic nude mice: tumor volume	miR-143, Bcl-2	(58)
Osteosarcoma	Upregulated	Oncogene	U2OS, SaOS2	Proliferation Invasion EMT	–	miR-641, YAP1	(69)
Osteosarcoma	Upregulated	Oncogene	U2OS/CDDP, SaOS-2/CDDP	Proliferation Invasion Migration Apoptosis Cisplatin resistance	–	miR-506-3p, SEMA6D, Wnt/β-catenin pathway	(70)
Osteosarcoma	Upregulate	Oncogene	KHOS/DXR, MG63/DXR	Proliferation Migration Invasion Apoptosis DXR Sensitivity	BALC/c nude mice: tumor volume, weight	miR-370, KLF12	(37)
Osteosarcoma	Upregulated	Oncogene	HOS, SaOS-2	Proliferation Migration Invasion Apoptosis	–	miR-204-3p, HMGA2	(71)
Osteosarcoma	Upregulated	Oncogene	SaOS-2, HOS	Proliferation Migration Invasion Apoptosis	Male nude mice: tumor volume, weight	miR-637, HMGB2	(72)
Gastric cancer	Downregulation	Suppressor gene	SGC-7901/CDDP, MKN-45/CDDP	Proliferation Apoptosis Cisplatin resistance	Male BALB/c mice: tumor volume, weight	miR-300, KAT6B	(59)
Clear cell renal cell carcinoma	Downregulated	Suppressor gene	786-O, A498, ACHN, Caki-1	Proliferation Migration Invasion Apoptosis	–	miR-148a-3p, FOXK2	(60)
Microcirculatory perfusion	Upregulated	–	PMVECs	Arterial pulsatility	Beagles	ZO-1, occludin, eNOS, NF-κB pathway	(44)
Microcirculatory perfusion	Upregulated	–	–	–	–	TNF-α, IL-1β, MCP-1, PI3K/Akt/mTOR pathway	(45)
Diabetic retinopathy	Upregulated	–	hRMECs	Viability Migration Tube formation	–	miR589-5p, EGR1	(35)
Osteoarthritis	Upregulated	–	CHON-001	Proliferation Migration Invasion Apoptosis Inflammation	–	miR-149-5p, WNT5B, IL-1β	(46)
Preeclampsia	Downregulated	–	HTR-8/SVneo, JEG-3	Proliferation Migration Apoptosis	–	miR-1244, FOXM1,	(47)
Milk fat metabolism	Upregulated	–	BMEC	–	Dairy cows	miR-331-3p	(48)

**Table 3:** Clinicopathological parameters of circ_0003945 in various cancers.

Tumor types	Role	Sample size of tumor tissue	Clinicopathological feathers	Statistical analysis	Ref.
Glioma	Oncogene	40	Overall survival (OS)	Kaplan–Meier survival analysis with the log-rank test	(61)
Papillary thyroid cancer	Oncogene	26	Lymph node metastasis	Fisher’s exact test	(63)
Non-small cell lung cancer	Oncogene	60	TNM stage, lymph node metastasis	Chi-square test	(43)
Triple-negative breast cancer	Oncogene	78	Tumor size, TNM stage, lymph node metastasis, OS	Chi-square test, Kaplan–Meier survival analysis	(34)
Luminal breast cancer	Oncogene	87	Histological type, tumor grade, molecular phenotype, OS, distant, metastasis-free survival (DMFS)	Chi-square test, Kaplan–Meier analysis with the log-rank test, Multivariate Cox proportional hazards analysis	(65)
Hepatocellular carcinoma	Oncogene	369	OS	Kaplan–Meier survival analysis	(52)
Hepatocellular carcinoma	Oncogene	30	Tumor size, tumor recurrence rate, OS, recurrence-free survival (RFS)	Chi-squared test, Kaplan–Meier survival analysis	(42)
Hepatocellular carcinoma	Oncogene	50	Tumor size, China liver cancer stage	Chi-square test	(41)
Hepatocellular carcinoma	Oncogene	20	Microvascular invasion, differentiation, OS, time to recurrence (TTR)	Chi-square test, Kaplan–Meier survival analysis, univariate and multivariate Cox proportional regression analyses	(66)
Pancreatic adenocarcinoma	Oncogene	126	OS	Kaplan–Meier survival analysis	(53)
Pancreatic ductal adenocarcinoma	Oncogene	128	Histological grade, lymph node metastasis, OS	Chi-square test, Kaplan–Meier survival analysis	(38)
Ovarian cancer	Oncogene	24	TNM stage, 5-year survival rate	Chi-square test, Kaplan–Meier survival analysis	(55)
Cervical cancer	Oncogene	58	OS	Kaplan–Meier survival analysis	(56)
Osteosarcoma	Oncogene	92	Tumor stage, OS	Spearman’s rank correlation assay, Kaplan–Meier survival analysis	(58)
Osteosarcoma	Oncogene	42	TNM stage, distant metastasis, survival rate	Chi-square test, Kaplan–Meier survival analysis	(71)
Osteosarcoma	Oncogene	40	Distant metastasis, TNM stage	Chi-square test	(72)
Gastric cancer	Suppressor gene	63	OS	Kaplan–Meier survival analysis	(59)

## 2 The biological functions and mechanisms of circ_0003945 in tumors

### 2.1 Biological functions of circ_0003945 in tumor cells

The expression of circ_0003945 was markedly elevated in tumor cell lines compared to the corresponding normal controls by quantitative real-time polymerase chain reaction (qRT-PCR) and statistical analysis of databases. Intriguingly, in gastric cancer and renal cell carcinoma cell lines, there was an aberrant downregulation of circ_0003945. The regulatory role of circ_0003945 in biological functions of tumor cells and potential mechanisms are as follows (**Table 2**).

#### 2.1.1 Proliferation and cell cycle

Cancer cells exhibit distinct metabolic processes compared to normal cells, characterized by reduced oxidative phosphorylation or abnormal aerobic glycolysis, which drive their growth and proliferation (73). Employing techniques involving cell counting Kit-8 (CCK-8), ethylenediurea (EDU), or colony formation assay, researchers have uncovered that the overexpression of circ_0003945 enhanced proliferation across mostly tumors cell lines (**Table 2**). The MAPK pathway comprises four primary branches: ERK, JNK, p38/MAPK, and ERK5, each playing a crucial role in the cells proliferation, differentiation, migration, and invasion of cancer cells (74). Notably, the upregulation of circ_0003945 suppressed mircoRNA (miR)–1244 and activated MAP3K2 and MAPK key factors (including ERK, JNK, and p38), thereby augmenting the proliferation of prostate cancer cells (57). Circ_0003945 knockdown reduced the proliferation marker proliferating cell nuclear antigen (PCNA) by performing as an miR-638 sponge. This action effectively sequestered miR-638 from SZRD1, resulting in restrain of glioma cell proliferation (39).

Deviation in the cell cycle progression is fundamental to tumor cell proliferation. The proceeding of the cell cycle is regulated by metabolic enzymes and upstream regulators, primarily through cyclin-dependent kinases (CDKs) and other critical regulators like APC/C or SCF E3 ligase complexes (75). Knockdown of circ_0003945 could block the progression of lung adenocarcinoma cells from the G1 to S phase by regulating the binding of p27 to the cyclin-CDK complex. This intervention led to cell cycle to arrest at G0/G1 phase, consequently diminishing cell proliferation *in vitro (* 51). This mirrored the function of circ_0003945 in glioma (62) and OC (68). Conversely, the upregulation of circ_0003945, acting as a tumor suppressor gene, impeded cell cycle in the G1 phase in clear cell RCC cells (60). Ki-67 acts as specific biomarker of cellular proliferation, regulated through cell cycle-dependent transcription and protein degradation processes (76, 77). Notably, overexpression of circ_0003945 was significantly associated with enhanced cellular of Ki-67 levels in NSCLC and HCC cells, implicating its roles in tumor cell proliferation (41, 43). *In vivo*, xenograft assays in mice had verified that stable knockdown of circ_0003945 led to reduced tumor volumes and weights compared matched controls. These findings indicate a promotive role for circ_0003945 in tumorigenesis, underlining its potential significance in cancer progression (**Table 2**).

#### 2.1.2 Apoptosis

Apoptosis, an evolutionarily conserved mechanism, plays a critical role in cellular turnover and tissue regeneration. A hallmark of this process is the release of cytochrome c from mitochondria. The regulatory framework of apoptosis involves a self-amplifying cascade among pro-apoptotic and anti-apoptotic proteins of the Bcl-2 family, along with the initiator caspases (caspase-8, -9, and -10) and downstream effector caspases (caspase-3, -6, and -7). In tumor cells, a disruption of this balance often triggers the apoptotic pathway, highlighting its potential as a target in cancer therapies (78, 79). Knockdown of circ_0003945 increased pro-apoptotic proteins Bax and caspase-3 and decreased anti-apoptotic protein Bcl-2, thereby inducing apoptosis in CC cells. It might exert functions by serving as a ceRNA for miR−361−3p, hampering SOX4 and thus impeding tumor cell progression (56). Knockdown of circ_0003945 reduced the expression of Bcl-2, conversely while elevating Bax and caspase-3 in OC cells. And overexpressing miR-382-5p could reverse this impact on apoptosis-related proteins and downstream PRPF8 gene (68). Furthermore, the study reported that, in NSCLC cell, silencing of circ_0003945 led to the downregulation of apoptosis-associated genes and proteins such as c-IAP1, Bcl-2, Survivin, and cell cycle protein CDK6, cyclin D1 were, while upregulation p27 and Bax (51). C-MYC is a multifunctional TF often associated with hepatocarcinogenesis. Its overexpression can enhance hepatocyte apoptosis (80). Previous studies indicated c-MYC as a crucial driver in transforming hepatocytes from the G0/G1 phase to the S phase (81). Overexpressing circ_0003945 notably increased the expression of c-MYC and cellular DNA synthesis, inhibited apoptosis in HCC. This mechanism accompanied by sponging miR-1294 (66).

#### 2.1.3 Autophagy

Autophagy is an essential catabolic process that recycles limited intracellular resources and mediates the degradation of damaged or redundant organelles under stress conditions to preserve cellular functionality (73). Previous studies have shown that epithelial cells can evade anoikis though autophagy and epithelial-mesenchymal transition (EMT), facilitating cell migration and invasion (82, 83). The accumulation of LC3B-II and the conversion from LC3B-I to LC3B-II is sensitive autophagy induction markers. In CRC, silencing circ_0003945 decreased total autophagosomes and autolysosomes formation. This knockdown impeded the expression of LC3B-II, lowered LC3B-I/II conversion rates, and decreased the degradation of key autophagy-related proteins like Beclin1, ATG7, and FOXO1. It was demonstrated that modulating circ_0003945 affected the miR-582-5p/FOXO1 axis, thereby inducing autophagy and promoting migration and invasion of CRC cells (40).

#### 2.1.4 Metastasis

The invasion-metastasis cascade, pivotal in cancer progression, involves extracellular matrix (ECM) degradation by a broad extent of cells and matrikines such as MMPs, Versican, and others. MMP-9, in particular, remodels the ECM, influencing tumor invasion, metastasis, and angiogenesis. The alteration of tumor stroma and release of angiogenic factors are key strategies in this process, supported by MMP-mediated vasculature growth (84–86). EMT is a cellular-transformed process marking by changes from E-cadherin–expressing epithelial cells to a mesenchymal phenotype expressing vimentin and N-cadherin, endowing tumor cells to migrate and invade (83, 87). In HCC cells, downregulating circ_0003945 enhanced E-cadherin and reduced N-cadherin and α-SMA levels, suggesting it EMT and invasion (52). Overexpression of circ_0003945 in NSCLC cells increased MMP9 and Fibronectin, reduced E-Cadherin via KLF4 and miR-3182, and activated Rac1/FAK1/MMP2 and JNK/MAPK pathways, promoting migration and invasion (51). In the glioma cell lines, downregulation of circ_0003945 brought on a diminished level of MMP9. But this effect could be reversed by miR-1205 or miR-382 depletion, indicating a regulatory role in cell migration and invasion (62). Furthermore, circ_0003945 dysregulated the Wnt/β-catenin pathway in HCC cells. It sponged miR-34c-5p to upregulate LGR4, activating β-catenin and accelerating migration. CHIR-9902127, a Wnt/β-catenin activator, showed reduced β-catenin phosphorylation in circ_0003945-knockdown HCC cells (41, 88). Additionally, sponging with miR-194-3p upregulated MMP9, a β-catenin target, facilitating HCC progression (42).

#### 2.1.5 Angiogenesis

Tumor cells often secrete high levels of pro-angiogenic factors which contribute to the development of an abnormal vascular network. However, the immaturity of tumor blood vessels impairs their functionality for the tumor microenvironment (TME) and increases risk of metastatic dissemination (89). Among these factors, VEGF, particularly VEGFA from the VEGF family, plays a pivotal role. VEGFA is crucial for cell proliferation, invasion, and angiogenesis in various malignancies (90). Circ_0003945 sponged miR-199a to upregulate VEGFA to promote CRC progression. And inhibiting miR-199a or overexpressing VEGFA could reverse the tumor-suppressing effects of circ_0003945 knockdown (54). Meanwhile, the tube formation assay in glioma cell lines revealed that knockdown of circ_0003945 led to reduced angiogenesis, evidenced by a decrease in branch formation. This suggested that circ_0003945 influenced the angiogenic capacity of glioma cells by the miR-638/SZRD1 axis (39).

#### 2.1.6 Immune escape

Malignant tumor cells often evade the surveillance of immune system, where immune cells can normally identify and eliminate malignancies. Tumor cells alter their structure, effect genes and protein expression within TME to evade immune surveillance, presenting a significant challenge to immunotherapy (91, 92). Cancer-associated fibroblasts (CAFs), prevalent in the TME, can contribute to this evasion. They aid tumor progression through ECM remodeling, growth factor production, cytokines and chemokines secretion, and metabolic and angiogenic modulation (93). In hepatitis virus-induced cirrhosis or liver cancer, CAFs originated from hepatic stellate cells and gradually transformed into a major ECM source due to the stroma cell accumulation. HCC-associated fibroblast implied CAF-derived CXCL11 enhanced cell invasion by regulating the circ_0003945/miR-4756/IFIT1/3 axis. Tumor cell morphology was altered from flattened to spindle-shaped and produced more pseudopods, causing elevated cell proliferation, DNA synthesis, migration ability, and protein levels of migration-related markers vimentin and twist (67). In pancreatic adenocarcinoma (PAAD), the circ_0003945/miR-494 axis regulated PAAD progression by CXCR4 and ZEB1, key mediators of tumor immune cell infiltration. Elevated CXCR4 and ZEB1 levels associated with immune cell markers and immune checkpoint proteins. This axis promotes M2 macrophage polarization of tumor-associated macrophages (TAMs), Treg recruitment and activation in TME, and induced T-cell depletion for immunological escape (53).

### 2.2 The potential molecular mechanisms of circ_0003945 regulating tumorigenesis

CircRNAs exert multiple biological functions, notably though the ceRNA mechanism mentioned upward. Previously described as the “Rosetta stone of a hidden RNA language,” ceRNA has garnered significant attention and undergone extensive research, bolstered by next-generation sequencing technology (94). CeRNAs cross regulate each other through sequestration of shared miRNAs and form complex regulatory networks based on their miRNA signature (95). Advances in sequencing technology have increasingly shown that ncRNAs, such as pseudogenes, small and long ncRNAs, and circRNAs, are key in biological processes and tumorigenesis (96, 97). MiRNAs are a short, single-stranded, and highly conserved class of 18- to 24-nt endogenous ncRNAs, undergo a multistep biogenesis process. Initiated by RNA pol II, the process produces primary-microRNAs (pri-miRNAs), which are cleaved into precursor-miRNA (pre-miRNA) by the Drosha-DGCR8 complex (98). Exported to the cytoplasm, these pre-miRNAs are further processed by ribonuclease Dicer into small double-stranded RNA (dsRNA) fragments. The functional strand is incorporated into the Argonaute (AGO) protein, forming the RNA-induced silencing complex (RISC). This complex functions as the primary effector in biological processes (99, 100). Mature miRNAs bind complementarily to the 3′untranslated region (3′UTR) of the target mRNA through miRNA response elements, either by base pairing or via additional sequence elements. AGO interacts with the polyA-tailed binding in the 3′end of mRNA by recruiting adapter protein TNRC6. This interaction facilitates post-transcriptional mRNA degradation and translation repression (101–103).

The mechanism of ceRNA involves endogenous transcripts, rich in miRNA-binding sites, that can remarkably dimmish the miRNA-mediated repression of target gene mRNA. CircRNAs, acting as sponges, compete with these miRNAs to sequester them from their original targets (97, 104–106). This forms a complex ceRNA network, where transcripts compete for miRNA, collaboratively modulating miRNA activity (107). Circ_0003945 networks link to the function of protein-coding mRNAs and related signaling pathways, demonstrating the extensive influence of these interactions (**Table 2**).

**Figure 4:**
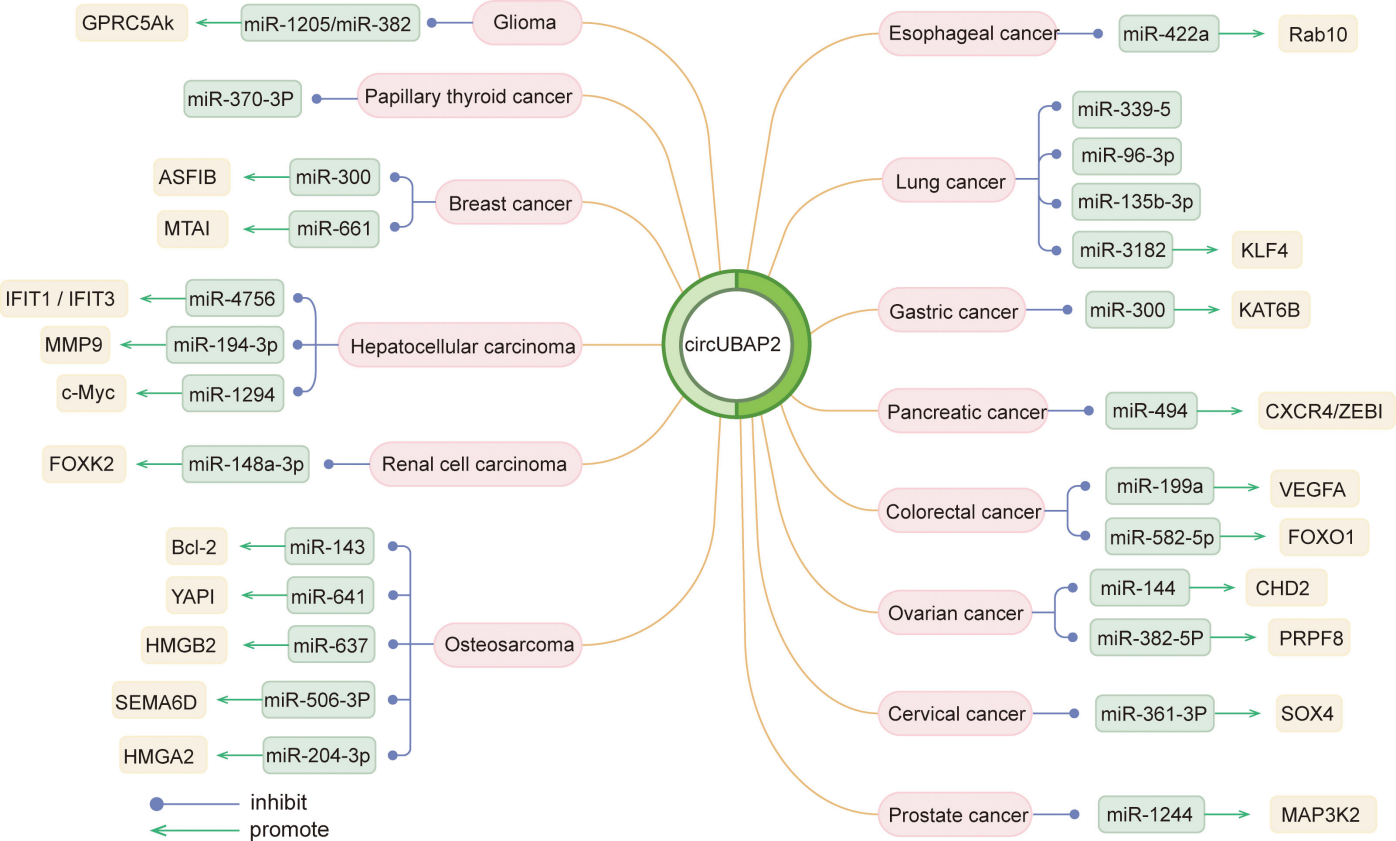
Molecular mechanisms of circ_0003945 in different diseases.

Circ_0003945 has been identified as a regulatory RNA, serving as a miRNA sponge. It specifically hampered miR-370-3p, with the inhibition of miR-370-3p reversing the effects of circ_0003945 on proliferation, apoptosis, and invasion in TC cells. Databases indicated the involvement of PI3K/Akt and Wnt pathway in TC regulation, with the PI3K/Akt/mTOR pathway being notably active in various cancers (63) (108). In cisplatin (DDP)-resistant triple-negative breast cancer (TNBC) cells, circ_0003945, functioning as a ceRNA for miR-300, upregulated ASF1B, thereby activating the PI3K/AKT/mTOR signaling and facilitating the DDP resistance to TNBC cells (64). The Hippo signaling is essential for cell growth and regeneration and works by phosphorylating YAP, with YAP1 being a key oncogenic transcriptional co-activator (109). In osteosarcoma, circ_0003945 targeted miR-641, which bond to YAP1’s 3′UTR, promoting cell proliferation, invasion, and EMT (69). Similar effects were observed in pancreatic ductal adenocarcinoma (PDAC), where circ_0003945 enhanced proliferation and migration through interacting with miR-6820-3p and promoting YAP1 expression (38). Analyses of circ_0003945-medicated ceRNA network in PAAD, via the Gene Expression Omnibus database, highlighted its influence on key pathways (NF-κB, PI3K-Akt, Wnt) and immune cell infiltration through the miR-494 axis and hub genes (CXCR4, HIF1A, ZEB1, and SDC1) (53). HMG (HMGB, HMGN, and HMGA) proteins are a family of nuclear proteins that bind to DNA, causing structural changes in chromatin (110). Circ_0003945 fostered aggressive behavior by sponging miR−204−3p to upregulate HMGA2 (71) and sponging miR-637 to upregulate HMGB2 (72) in osteosarcoma cells. And in esophageal squamous cell carcinoma (50) and OC (55), it promoted cancer cell behavior via the miR-422a/Rab10 and miR-144/CHD2 axes, respectively. Overall, the circRNA-miRNA-mRNA regulatory network involving circ_0003945 underscores its crucial role in disease mechanisms (**Figure 4**).

## 3 The clinical significance and prognostic value of circ_0003945 in tumors

Overexpression of circ_0003945 tended to mean more aggressive clinicopathological parameters and poorer prognosis for tumor patients in major conditions. Its upregulation was more prone to increased therapy resistance through multiple mechanisms.

### 3.1 The clinical parameters of circ_0003945 in tumors

Numerous studies have identified circRNAs as potential diagnostic biomarker in cancers (12). Specifically, circ_0003945 expression was found to be higher in various cancer tissues compared to paired adjacent noncancerous tissues, though it is notably decreased in gastric cancer and renal cell carcinoma. (**Table 2**). High circ_0003945 levels correlated with aggressive clinicopathological characteristics such as invasive histological types, poor differentiation, higher recurrence rates, extensive microvascular invasion, and advanced tumor-node-metastasis (TNM) stages, including larger tumor size, more lymph node and distant metastases (**Table 3**). Kaplan–Meier survival analysis showed that patients with high circ_0003945 levels had shorter overall survival (OS), indicating a poorer prognosis (**Table 3**). Moreover, higher circ_0003945 expression was linked to reduced recurrence-free survival (RFS) and is an independent predictor of time to recurrence (TTR) in hepatocellular carcinoma. In tamoxifen-resistant patients, elevated circ_0003945 levels were associated with worse distant metastasis-free survival (DMFS) (65).

Currently, there are no related clinical trials targeting circ_0003945 in human diseases. However, some other clinical trials targeting circRNAs have been carried out so far. For example, studies identified and compared differentially expressed of relevant circRNAs in various cells or tissues. Independent cohorts confirmed the potential of certain circRNAs as sensitive and specific biomarkers for diseases diagnosis and prognosis prediction (111–114).

### 3.2 Circ_0003945-related therapeutic resistance

Cancer therapeutic resistance is broadly classified into intrinsic and acquired (including adaptive) types, with tumor cells traditionally categorized as either drug-sensitive or drug-resistant. Resistance often involves pathway-based mechanisms, with activation of drug-inhibited effector proteins upstream, parallel, or downstream of the primary targets (115). The upregulation of circ_0003945 has been linked to augmented cisplatin resistance in TNBC cells. Circ_0003945 curbed TNBC sensitivity to cisplatin through the miR-300/ASF1B axis by activating PI3K/AKT/mTOR pathway (64). Knockdown of circ_0003945 hampered SEMA6D to reversing cisplatin resistance via sponging miR-506-3p by restraining the Wnt/β-catenin signaling pathway in osteosarcoma (70). Conversely, as a tumor suppressor gene in gastric cancer, circ_0003945 hindered cisplatin resistance through the miR-300/KAT6B axis (59). Additionally, the depletion of circ_0003496 suppressed tumor growth and enhanced doxorubicin (DXR) sensitivity in osteosarcoma by targeting KLF12 via miR-370 (37). In NSCLC, its downregulation significantly mitigated resistance to docetaxel, DXR, and gefitinib, via restricting KLF4 through modulation of miR-3182 (43). Furthermore, research discovered a potential mechanism in tamoxifen resistant, where circ_0003945 acting though miR-1296-5p/STAT1 axis, contributed to tamoxifen-resistant in luminal breast tumors (65). In glioma cells, inhibiting circ_0003945 increased radiosensitivity by weakening RNF2 and counteracting the effects of miR-433-3p (61).

Generally, the upregulation of circ_0003945 was consistently associated with therapeutic resistance by signaling pathways in drug-resistant cancer cell lines and tissues (37, 43, 59, 61, 64, 65, 70). It might provide novel perspective on reserving drug resistance or improving radiosensitivity in tumor therapy.

## 4 Role of circ_0003945 in non-malignant conditions

In cardiovascular disease, circ_0003945 was correlated with microcirculatory perfusion. It inhibited the migratory activity and promoted M2 polarization in macrophages, declining the productions of cytokines TNF-α, interleukin (IL)-1β, and MCP-1 and the PI3K/AKT/mTOR pathway.

The expression of circ_0003945 might predict prognosis in extracorporeal membrane oxygenation (ECMO) patients with cardiogenic shock (45). In a canine ECMO model, the modifications of pulsatility improved microcirculatory perfusion and endothelial integrity. The upregulation of circ_0003945 stabilized endothelial tight junction markers ZO-1 and occluding. It followed by modulating the eNOS activity and inhibiting the NF-κB signaling pathway, pivotal in this protective mechanism (44).

Furthermore, circ_0003945 was upregulated in DR, when knocked down, it alleviated high glucose-triggered oxidative stress and dysfunction in human retinal microvascular endothelial cells (hRMECs). It might offer a promising therapeutic target through the miR589-5p/EGR1 axis for DR (35). In OA, exosome-mediated circ_0003945 participated in IL-1β–induced chondrocyte damage. It stimulated WNT5B via hindered miR-149-5p, affecting chondrocyte proliferation, apoptosis, migration, invasion, inflammation, and ECM degradation (46). Circ_0003945 was found to be downregulated in placental tissues from patients with PE compared to healthy controls. Its knockdown impeded trophoblast cell proliferation and migration via miR-1244/FOXM1 axis (47). Interestingly, circ_0003945 was significantly upregulated in cow mammary gland tissue, influencing milk fat metabolism by sponging miR-331-3p (48). These studies underscore the potential role of circ_0003945 in various diseases beyond tumors.

## 5 Conclusions and prospects

CircRNAs have garnered widespread attention in contemporary medical research due to their indispensable roles in human diseases pathogenesis. In this review, we discussed a compendium of studies that document the aberrant expression of circ_0003945 in various human diseases. These studies elucidate the specific cellular and biological functions of this circRNA. In most malignancy cases, circ_0003945 served as an oncogene. It functioned as a miRNA sponge, inhibiting the transcription of miRNAs, and thereby activates downstream effector genes. However, in a minority of malignancies, such as GC and RCC, circ_0003945 acted as a tumor suppressor gene. Research has indicated that circ_0003945 could alter the biological behaviors of tumor cells by modulating signaling pathways such as MAPK, Wnt/β-catenin, PI3K/AKT/mTOR, Rac-FAK1, and others. The expression levels of circ_0003945 were strongly correlated with the clinicopathological features of patients, and survival analyses also provided valuable insights into prognosticating clinical outcomes. Moreover, circ_0003945 affected sensitivity to chemotherapy and radiotherapy through alterations in its molecular mechanisms. This finding indicated that circ_0003945 might serve as a valuable predictive biomarker for clinical management of diseases.

Nonetheless, current research on circ_0003945 remands relatively restricted. The primary issue is the obscurity surrounding the upstream regulatory mechanisms of circ_0003945. Within the TRCirc database, the TF-peak of SE FOXA1 is located squarely in the transcriptional domain of circ_0003945. Further empirical validation is needed to determine whether a specific TF can directly and independently regulate the transcription of circ_0003945, thereby impacting the function of downstream proteins. Additionally, most molecular mechanisms identified for circ_0003945 are limited to the model of ceRNA, leaving other functionalities of circRNAs yet to be elucidated. Moreover, there is an insufficient exploration of the correlations with unmentioned sub-histological even pathological types of tumors in experiments. Furthermore, significant advancements in clinical trials, including consecutive biopsies of tumor tissues and circulating plasma sampling, are essential to definitively ascertain whether circ_0003945 can serve as a reliable clinical diagnostic maker. Finally, when relevant clinical trials concerning circ_0003945 are conducted and yield valid conclusions, the potential of circ_0003945 as a target for clinical therapy is expected to be realized.

## Author contributions

XZ: Writing – original draft, Conceptualization, Investigation, Visualization. LM: Writing – original draft, Conceptualization, Funding acquisition, Investigation, Writing – review & editing. LW: Writing – original draft, Conceptualization, Visualization, Writing – review & editing. HW: Visualization, Writing – original draft, Conceptualization. ZW: Funding acquisition, Project administration, Supervision, Writing – review & editing, Conceptualization.

## Glossary

ncRNA: non-coding RNA
circRNA: circular RNAs
ceRNA: competitive endogenous RNA
HDV: hepatitis delta virus
pol II: polymerase II
TF: transcription factor
TAH: transcriptionally activated to a higher level than the host genes
SE: super enhancer
iRegulon: the intragenic regulon
nt: nucleotide
EcircRNAs: exonic circRNAs
CiRNAs: intronic circRNAs
EIciRNAs: exon–intron circRNAs
tricRNAs: tRNA introns cirRNAs
miRNA: microRNA
miR: mircoRNA
snRNP: small nuclear ribonucleoprotein
UBAP2: ubiquitin-associated protein 2
RNase R: ribonuclease R
DR: diabetic retinopathy
OA: osteoarthritis
PE: preeclampsia
TC: thyroid cancer
EC: esophageal cancer
NSCLC: non-small cell lung cancer
BC: breast cancer
TNBC: triple-negative breast cancer
HCC: hepatocellular carcinoma
CRC: colorectal cancer
OC: ovarian cancer
CC: cervical cancer
GC: gastric cancer
RCC: renal cell carcinoma
PAAD: pancreatic adenocarcinoma
PDAC: pancreatic ductal adenocarcinoma
qRT-PCR: quantitative real-time polymerase chain reaction
CCK-8: cell counting Kit-8
EDU: ethylenediurea
PCNA: proliferating cell nuclear antigen
CDK: cyclin-dependent kinase
EMT: epithelial-mesenchymal transition
ECM: extracellular matrix
TME: tumor microenvironment
CAFs: cancer-associated fibroblasts
TAMs: tumor associated macrophages
pri-miRNAs: primary-microRNAs
pre-miRNAs: precursor-miRNAs
dsRNA: double-stranded RNA
AGO: Argonaute
RISC: RNA-induced silencing complex
3′UTR: 3′ untranslated region
DDP: diamminedichloroplatinum/cisplatin
GEO: Gene Expression Omnibus
TNM: tumor-node-metastasis
OS: overall survival
RFS: recurrence-free survival
TTR: time to recurrence
DMFS: distant metastasis-free survival
DXR: doxorubicin
IL: interleukin
ECMO: extracorporeal membrane oxygenation
hRMECs: human retinal microvascular endothelial cells
MAPK: mitogen-activated protein kinase
ERK: extracellular signal-regulated kinase
JNK: jun N-terminal kinase
FGF9: fibroblast growth factor 9
APC/C: anaphase-promoting complex/cyclosome
SCF: SKP1-Cullin-F-box
SOX4: SRY-related high-mobility-group box 4
c-IAP1: cellular inhibitors of apoptosis 1
LC3B: light chain 3B
ATG: autophagy-related
FOXO1: forkhead box transcription factor O1
MMPs: matrix metalloproteinases
α-SMA: alpha-smooth muscle actin
KLF4: kruppel-like factor 4
RAC1: ras-related C3 botulinum toxin substrate 1
FAK: focal adhesion kinase
LGR4: leucine-rich repeat-containing G protein–coupled receptor 4
VEGF: vascular endothelial growth factor
CXCL: C-X-C chemokine ligand
CXCR: C-X-C chemokine receptor
ZEB1: zinc finger E-box binding homeobox 1
HIF: hypoxia-inducible factor
SDC1: syndecan-1
IFIT: tetratricopeptide repeats
DGCR8: DiGeorge syndrome critical region gene 8
TNRC6: trinucleotide repeat containing 6
PI3K: phosphatidylinositol-3 kinase
mTOR: mammalian target of rapamycin
YAP: yes-associated protein
HMG: high-mobility group
CHD2: chromodomain helicase DNA binding protein 2
ASF1B: anti-silencing function 1B
SEMA6D: semaphorin 6D
KAT6B: lysine acetyltransferase 6B
STAT1: signal transducer and activator of transcription 1
RNF2: ring finger protein 2
eNOS: endothelial nitric oxide synthases
TNFα: tumor necrosis factor α
MCP-1: monocyte chemoattractant protein- 1
EGR1: early growth response factor 1
WNT5B: wingless-type MMTV integration site family, member 5B
FOXM1: forkhead box protein M1
